# The cost-effectiveness of changes to the care pathway used to identify depression and provide treatment amongst people with diabetes in England: a model-based economic evaluation

**DOI:** 10.1186/s12913-017-2003-z

**Published:** 2017-01-24

**Authors:** Ben Kearns, R. Rafia, J. Leaviss, L. Preston, J.E. Brazier, S. Palmer, R. Ara

**Affiliations:** 1 School of Health and Related Research, University of Sheffield, Sheffield, UK S1 4DA; 2 Centre for Health Economics, University of York, Heslington York, UK YO10 5DD

**Keywords:** Diabetes mellitus, Depression, Health economics, Mass screening, Collaborative care

## Abstract

**Background:**

Diabetes is associated with premature death and a number of serious complications. The presence of comorbid depression makes these outcomes more likely and results in increased healthcare costs. The aim of this work was to assess the health economic outcomes associated with having both diabetes and depression, and assess the cost-effectiveness of potential policy changes to improve the care pathway: improved opportunistic screening for depression, collaborative care for depression treatment, and the combination of both.

**Methods:**

A mathematical model of the care pathways experienced by people diagnosed with type-2 diabetes in England was developed. Both an NHS perspective and wider social benefits were considered. Evidence was taken from the published literature, identified via scoping and targeted searches.

**Results:**

Compared with current practice, all three policies reduced both the time spent with depression and the number of diabetes-related complications experienced. The policies were associated with an improvement in quality of life, but with an increase in health care costs. In an incremental analysis, collaborative care dominated improved opportunistic screening. The incremental cost-effectiveness ratio (ICER) for collaborative care compared with current practice was £10,798 per QALY. Compared to collaborative care, the combined policy had an ICER of £68,017 per QALY.

**Conclusions:**

Policies targeted at identifying and treating depression early in patients with diabetes may lead to reductions in diabetes related complications and depression, which in turn increase life expectancy and improve health-related quality of life. Implementing collaborative care was cost-effective based on current national guidance in England.

**Electronic supplementary material:**

The online version of this article (doi:10.1186/s12913-017-2003-z) contains supplementary material, which is available to authorized users.

## Background

Diabetes is a chronic condition associated with premature death and a number of serious complications such as amputation, blindness and heart disease [1, 2]. Self-management plays an important role in the treatment of diabetes, however the presence of comorbid depression is associated with poorer self-management, leading to reduced adherence to medication and poorer glycaemic control [3]. The effects of comorbid depression on outcomes for people with diabetes are marked; mortality is increased by over a third, the prevalence of complications is increased, and healthcare costs are 4.5 times higher [4–6].

Historically services of care in the English National Healthcare System (NHS) have been based around single-disease guidelines, leading to “siloing” of care [7, 8]. However, in recent years there has been growing interest in exploring methods by which the care pathways for people with comorbid physical and mental health problems may be integrated, and in assessing the impact that any resulting changes have on both patient outcomes and costs to the healthcare system [4]. There is evidence that addressing comorbid depression amongst people with diabetes can lead to improved outcomes and quality of life [4]. For example, one study showed that implementing a 12-month depression treatment program for people with diabetes led to reduced outpatient resource use [9].

Potential enhancements to the care pathway for individuals with diabetes and depression include implementing collaborative care, and improving rates of opportunistic screening. Collaborative care is an enhancement to how depression treatment is usually delivered. It requires an additional healthcare professional, whose job is to improve collaboration between the individual receiving depression treatment and those delivering the depression treatment [10]. It is recommended in English National Institute for Health and Care Excellence (NICE) clinical guidelines for individuals with depression and a long-term chronic physical health problem [11], and has been shown to be effective in both the United Kingdom (UK) [12] and the United States [13]. However, it is not yet routinely implemented within the UK. Opportunistic screening refers to screening for depression amongst routine primary care appointments unrelated to depression. One method by which rates of opportunistic screening may be improved is as part of a policy to screen individuals with diabetes for depression during every primary care appointment.

This study aimed to assess the cost-effectiveness of three potential service changes to the current pathways of care for individuals with both diabetes and depression. These service changes were: implementing collaborative care, improving opportunistic screening, and combining collaborative care with improved opportunistic screening.

## Methods

A mathematical model was developed to assess the cost-effectiveness of potential changes (policies) to the current care pathways (standard care) experienced by people diagnosed with type-2 diabetes (T2DM) currently managed within primary care in England. Patients could have existing depression, develop depression, or remain depression free. A group of expert advisors assisted in the identification of both relevant service changes and relevant evidence.

### Model structure

**Fig. 1:**
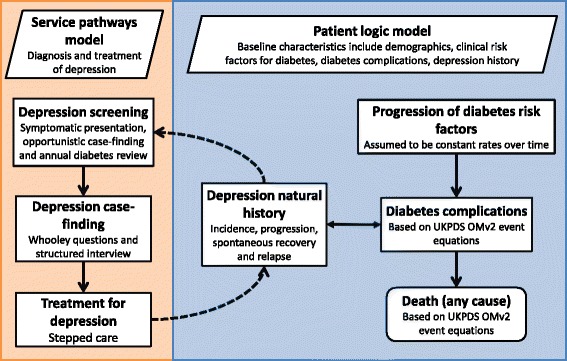
Simplified model schematic

The patient-level mathematical model used discrete-event simulation, and consisted of two inter-linked disease-specific models for diabetes and depression. A simplified schematic of the model is presented in Fig. 1.

The diabetes sub-model, which included mortality, was a modification of the United Kingdom Prospective Diabetes Study Outcomes Model version 2 (UKPDS OMv2), which has been described in detail elsewhere [14]. Briefly, this used patient characteristics (demographics and clinical measurements) to assess the progression of diabetes risk factors over time, and the development of diabetes-related complications. Diabetes-related complications included were either microvascular (ischaemic heart disease [IHD], blindness or renal failure) or macrovascular (heart failure, myocardial infarction [MI], stroke, diabetes ulcer or amputation). Both the risk of developing a diabetes-related complication and the probability of dying were taken from published regression models from the UKPDS OMv2, based on an individual with T2DM’s diabetes-related risk factors for complications and history of other complications. Progression rates for diabetes-related risk factors for complications included in the UKPDS OMv2 have not been published and hence were assumed to be constant. This is similar to the approach used in other diabetes models [15].

The depression sub-model was based on an existing model of depression care pathways, which was adapted to include minor (sub-threshold) depression as a separate health state [16]. This sub-model included the natural history of depression in the absence of treatment (in the form of incidence, remission and recurrence), and the impact of depression screening and treatment on this natural history.

The two disease-specific models were linked by modelling a bi-directional relationship between depression and diabetes-related complications such that individuals with T2DM and diabetes-related complications were more likely to experience depression; whilst patients with depression were more likely to develop diabetes-related complications. Successful depression treatment had the potential to decrease the time to remission. Conversely, the development of a diabetes-related complication had the potential to increase the hazard of depression incidence or recurrence.

### Model inputs

Model parameters were informed by literature reviews, as described elsewhere [1]. Where possible, evidence was taken from studies that considered patient populations with both diabetes and depression. Otherwise, data from patients with depression alone or patients with diabetes irrespective of depression status were used supplemented by expert opinion. The evidence for depression progression, screening and case-finding used in the economic model is summarised in Table 1, whilst the evidence for depression treatment is summarised in Additional file 1. Evidence used to inform the progression of diabetes risk factors and the development of diabetes related complications were taken from the UKPDS OMv2 [14].

**Table 1:** Summary of parameters used for depression progression, screening and case-finding

Parameters	Value	Source
Incidence of depression (per year) in patients with diabetes and no history of depression		
Minor	5.4%	Assumption
Major	5.4%	Nefs et al. [20]
Time to progression (years)		
Minor to major depression	42% at 2 years	Bot et al. [19]
Time to relapse of depression for patients with a history of depression (years)		
Minor	1.359	Assumption
Major	1.359	Lutsman et al. [21]
Time to spontaneous recovery (years)		
Minor	0.354	NICE CG90 [24]
Major	0.877	Spijker et al. (2002) [38]
Average annual number of GP appointments (other than appointments associated with depression treatment)		
• cigDiabetes, no depression • Diabetes with minor depression • Diabetes with major depression	12.5 8 8	Bhattarai et al. [22] Assumption Assumption
Probability that a GP appointment includes a depression screen		
• No history of depression • History of depression	5% 20%	Assumption
Probability of attending annual diabetes review		QOF 2012/13 [39] DM29
• No depression • Minor depression • Major depression	90.4% RR^a^: 0.9 RR^a^: 0.65	Assumption Assumption
Probability that the annual review includes a depression screen	85.9%	QOF 2012/13 [39] DEP1
Effectiveness of screening		
• Sensitivity of Whooley questions • Specificity of Whooley questions • Sensitivity of structured interview • Specificity of structured interview	95% 66% 100% 100%	NICE CG91 [11] NICE CG91 [11] Assumption Assumption

^a^ *RR* Relative risk. Values are relative to no depression, with values <1 indicating that patients are less likely to attend the annual diabetes review. *QOF* Quality and outcomes framework

Baseline characteristics were taken mainly from the National Diabetes Audit [17] and the UKPDS OMv2 [14]. Neither of these contained evidence on the prevalence of depression amongst individuals with T2DM; evidence for major depression was taken from a cross-sectional English study [18]. Further details about the baseline characteristics are provided in Additional file 1. The rate of progression from minor to major depression was based on the randomised-controlled trial of Bot et al. [19]. Evidence about the incidence of major depression and relapse rates amongst individuals with diabetes were taken from a Dutch cohort study [20] and a randomised-controlled trial [21] respectively. In the absence of suitable evidence, the incidence of, prevalence of, and relapse rates for minor depression were assumed to be the same as those for major depression.

The average annual number of routine primary care appointments for individuals with T2DM but no depression was taken to be 12.5, based on published evidence [22]. It was assumed that individuals with both T2DM and depression would be less likely to engage with healthcare services and so have an average of eight primary care appointments per annum (excluding any appointments relating to their depression treatment). It was presumed that under the current care pathways, individuals with T2DM and a history of depression had a 20% probability of receiving an opportunistic screen during a primary care appointment, and a 5% probability for individuals with T2DM and no history of depression. It was assumed that the Whooley questionnaire was used for depression screening, with sensitivity and specificity taken from an existing meta-analysis [11]. The choice of the Whooley questionnaire for depression screening was motivated by national guidance in the UK Quality and Outcomes Framework [23]. Advisors concurred with this approach, although they noted that in practice the Whooley questions may be asked formally or informally. Patients with a positive screen underwent depression case-finding in the form of a structured interview, which was assumed to either confirm cases of depression (amongst people with depression) or correct cases of incorrectly identified depression (amongst people without depression).

Evidence on the effectiveness of depression treatment was taken from meta-analyses for both pharmacotherapy [24] and low-intensity psychotherapy [25]. Evidence for high-intensity psychotherapy was taken from an existing economic evaluation [16], who considered depression amongst the general population.

### The bi-directional association between depression and diabetes-related complications

Based on the available evidence [1] and discussions with advisors, it was assumed that there was a bi-directional association between depression and diabetes-related complications, such that:

- Individuals with T2DM and depression had a higher risk of developing diabetes-related complications
- Individuals with T2DM and diabetes-related complications had a higher hazard of developing depression.

This association was incorporated within the model via a set of hazard ratios. Individuals with T2DM had an annual probability of developing a diabetes-related complication based on the UKPDS risk equations [14]. If an individual with T2DM had depression this risk was elevated in the model, based on the amount of time spent with depression in the preceding year (for example, if an individual spent half of the year with depression, then the elevated risk due to having depression was halved). Further, if an individual with T2DM and depression was receiving depression treatment, and they ultimately responded to it, it was assumed that their time spent with depression did not contribute towards an elevated risk. The hazard ratios for developing a diabetes-related complication due to having depression were taken from Lin et al. [26], for microvascular disease they were 1.31 for minor depression and 1.36 for major depression, whilst for macrovascular disease they were 1.00 for minor depression and 1.25 for major depression (values greater than one indicate an increased risk). If an individual with T2DM had a diabetes-related complication, the hazard for developing depression was increased. It was assumed that the hazard of developing depression was the same if individuals had one or several complications. No studies were identified which estimated the effect of having a diabetes complication on developing depression, so a hazard ratio of 1.5 was assumed for the effect of any diabetes complication on either minor or major depression. Further details on how this association was modelled are provided in Additional file 1. The robustness of model results to this assumed value was tested in sensitivity analyses.

### Health-related quality of life and costs

The baseline utility values (measured using the EQ-5D) for patients with diabetes and the decrements in health-related quality of life (HRQoL) associated with MI, IHD, stroke, heart failure, amputation and blindness were taken from the most recent analysis of the UKPDS [27]. Utility decrements associated with foot ulcers, severe hypoglycaemia and renal failure were not reported in this study and were sourced from the literature [28–30]. No studies were identified which reported on the impact of depression on HRQoL for patients with diabetes. These utility decrements were derived from a study amongst patients with depression [31]. It was assumed that the utility decrement due to depression was halved for the duration of treatment if the individual ultimately responded to treatment, reflecting the concept that HRQoL improved whilst on successful treatment. Estimates of resource use and unit costs were taken from national sources and from the literature [16, 32]. An overview of the costs and utilities is provided in Table 2, with details about resource use for depression treatment available in Additional file 1 (section B). In general, resource use and costs for depression treatment came from the economic evaluation of Tosh et al. [16]. The costs of treating diabetes and its complications were taken from Clarke et a.l [32]. Remaining costs were primarily based on national sources or assumptions where evidence was lacking. Based on discussions with advisors, it was unclear if implementing an opportunistic screen for depression would lead to an increase in the costs of a GP appointment. For the base-case an additional cost of £2 was used; two sensitivity analyses considered having no additional cost, and an additional cost of £4. Further detail on how the costs were derived is available elsewhere [1].

**Table 2:** Costs and utilities used in the economic model

Unit costs		
GP appointment	£37	PSSRU [40]
Annual review	£397	NAO [41]
Opportunistic screening for depression	£2	Assumption
Antidepressants (daily costs)	£0.073	Tosh et al. [16]
IAPT per session	£88	Tosh et al. [16]
Diabetes-related complications^*^		
Diabetes — no complications	£252	Clarke et al. [32]
CHF — year of event / subsequent years	£3,559 / £1,01	Clarke et al. [32]
IHD — year of event / subsequent years	£3,139 / £790	Clarke et al. [32]
MI — year of event / subsequent years	£6,522 / £744	Clarke et al. [32]
Stroke — year of event / subsequent years	£3,793 / £399	Clarke et al. [32]
Blindness — year of event / subsequent years	£1,397 / £450	Clarke et al. [32]
Ulcer — year of event / subsequent years	£1,855 / £21	Ghatnekar et al. [42]
Amputation — year of event / subsequent years	£13,556 / £481	Clarke et al. [32]
Renal failure — year of event / subsequent years	£34,806 / £34,806	NICE STA for dapagliflozin [43]
Severe hypoglycaemia	£390	NICE STA for dapagliflozin [43]
Health state utilities	Value	References
Baseline	0.807	Alva et al. [27]
Decrements		
MI (year before)	−0.065	Alva et al. [27]
MI (prior history)	0.008	Alva et al. [27]
IHD	−0.028	Alva et al. [27]
Stroke	−0.165	Alva et al. [27]
Heart Failure	−0.101	Alva et al. [27]
Amputation	−0.172	Alva et al. [27]
Blindness	0.033	Alva et al. [27]
Renal failure	−0.263	Klarenbach et al. [29]
Foot ulcer	−0.016	Sollie et al. [28]
Severe Hypoglycaemia	−0.00186	Marrett et al. [30]
Minor depression	0	Kaltenthaler et al. [31]
Major depression	−0.3	Kaltenthaler et al. [31]
Effect of being on depression treatment on the decrement for major depression; responders only (multiplier)	x0.5	Assumption

^*^Uplifted to 2013 prices

*GP* General practitioner, *MI* myocardial infarction, *IHD* ischaemic heart disease

### Policy changes modelled

The policy of implementing collaborative care was modelled as an enhancement to the existing care pathway for individuals with depression and diabetes. The additional resource use associated with collaborative care was based on resource use patterns described in NICE clinical guideline 91 [11]. Implementing collaborative care was assumed to decrease the probability of drop-out (relative risk 1.33) and increase the probability of responding to depression treatment (relative risk 1.79), with evidence taken from a meta-analysis by Huang et al. [33]. Further details on the implementation of collaborative care are available in Additional file 1 (section C).

Under the policy of improved opportunistic screening, it was assumed that every primary care appointment for individuals with diabetes included an opportunistic screen for depression (with costs as detailed in Table 2), unless the individual had identified depression.

The combined policy included the costs and effects for both collaborative care and improved opportunistic screening, with no further changes.

### Assessment of cost-effectiveness

Health economic outcomes were summarised using the incremental cost effectiveness ratio (ICER), defined as incremental costs divided by incremental quality adjusted life years (QALYs). The clinical outcomes considered were the number of diabetes-related complications and the number of depressive episodes (identified and unidentified) experienced by an individual with T2DM. The analysis took the perspective of the NHS and social services. Wider societal benefits were also explored which considered the impact on productivity (days off work due to ill-health) and informal care (days received any unpaid care provided by family or friends). This was based on methodology developed to inform the Department of Health’s proposed approach to Value Based Pricing [34].

Costs were reported in 2013 British Pound Sterling. A lifetime horizon was used. Both costs and QALYs were discounted at a rate of 3 · 5 per cent per year [35]. All results were estimated by running 600,000 patients through the mathematical model, and multiplying-up the results to reflect those for a cohort of 2,000,000 adults diagnosed with T2DM in England [17].

### Assessment of uncertainty

A Probabilistic sensitivity analysis was not performed due to computational restrictions. Thirty univariate sensitivity analyses were conducted by changing the values of specified model inputs and assessing the impact on the health economic results. These sensitivity analyses included changes to the natural history of depression, the bi-directional association between having a diabetes-related complication and depression, the magnitude of disutility due to having depression, and the costs of screening and collaborative care. Further details are available in Additional file 1.

## Results

### Number of diabetes-related complications and depressive episodes

Predicted lifetime clinical outcomes experienced by the cohort for current practice and each of the policy changes are displayed in Table 3. Under current practice the cohort would experience approximately 1.5 million diabetes-related complications. All three policy changes produced a reduction in both microvascular and macrovascular diabetes-related complications. The combined policy (collaborative care and improved opportunistic screening) avoided the greatest proportion of complications; about 1.5%, whilst the other two policies both avoided about 1%.

**Table 3:** Lifetime incidence of diabetes-related complications and depression episodes

Results per 2,000,000 people	Current practice (CP)	Policy 1^a^	Policy 2^b^	Policy 3^c^	Policy 1 — CP	Policy 2 — CP	Policy 3 — CP
Number of microvascular complications							
Blindness	116,237	114,360	115,070	113,287	−1,877	−1,167	−2,950
Renal failure	68,633	66,840	66,890	66,150	−1,793	−1,743	−2,483
Diabetic ulcer	69,320	68,803	68,633	67,920	−517	−687	−1,400
Amputation	87,017	85,303	85,450	84,560	−1,713	−1,567	−2,457
Total	341,207	335,306	336,043	331,917	−5,900	−5,164	−9,290
Number of macrovascular complications							
IHD	277,880	275,627	276,923	274,883	−2,253	−957	−2,997
MI	377,417	374,227	374,190	373,437	−3,190	−3,227	−3,980
Stroke	274,880	273,010	272,980	271,650	−1,870	−1,900	−3,230
CHF	221,130	219,640	219,250	218,587	−1,490	−1,880	−2,543
Total	1,151,307	1,142,504	1,143,343	1,138,557	−8,803	−7,964	−12,750
Number depression episodes (1,000)							
Total number	15,517	15,598	15,563	15,605	80	46	88
Identified	7,937	7,983	13,547	13,588	46	5,610	5,650
Unidentified	7,580	7,615	2,016	2,018	35	−5,564	−5,562

*CP* Current practice, *IHD* Ischaemic heart disease, *MI* Myocardial infarction, *CHF* Congestive heart failure. ^a^Policy 1 = collaborative care; ^b^Policy 2 = opportunistic screening; ^c^Policy 3 = both collaborative care and opportunistic screening

It was predicted that the cohort would experience about 15.5 million depressive episodes under current clinical practice over their lifetime, of which just over half (51%) would be identified. Implementing collaborative care did not affect the proportion of identified depression episodes, as this service change only affects depression treatment. However, implementing improved opportunistic screening, either on its own or in combination with collaborative care was estimated to increase the proportion of identified cases to approximately 87%.

### Health economic outcomes

Results of the cost-effectiveness analysis are provided in Table 4. Of the three service changes considered, the combined policy was estimated to have the largest impact on both life years and QALYs compared with current practice, with increases of 0.6 and 1.8% respectively. These increases were driven by a reduction in the number of diabetes related complications. The largest reductions were observed for renal failure and amputation, which also have the largest impact on HRQoL. However, this policy was also associated with the largest increases in lifetime healthcare costs, with an increase compared to current practice of £9.6 billion (23.2%) from £41.6 billion. Increases for the other policies were: collaborative care £6.7 billion (16.2%) and improved opportunistic screening £1.6 billion (3.9%). The main drivers for increased costs were an increase in the costs of treating depression, and an increase in the costs of the diagnostic interview (for the two policies that included changes in opportunistic screening).

**Table 4:** Cost-effectiveness results

Discounted results (Results per 2,000,000 people)	Current practice (CP)	Policy 1^a^	Policy 2^b^	Policy 3^c^	Policy 1 — CP	Policy 2 — CP	Policy 3 — CP
Life years (1,000)	19,515	19,580	19,564	19,601	65	49	86
QALYs (1,000)	12,006	12,103	12,082	12,188	97	76	182
Informal care (1,000)	4,975	4,947	4,953	4,898	−27	−22	−77
Days off sick (100)	1,733	1,705	1,711	1,673	−27	−21	−60
QALY loss due to depression (1,000)	1,746	1,695	1,702	1,631	−51	−44	−115
Costs (2013, £1,000,000)	29,626	30,676	34.475	36,431	1,050	4,849	6,805
Costs (2013 UK £1,000,000) Undiscounted							
Complications management	9,833	9,644	9,459	9,428	−190	−374	−405
Annual review	9,134	9,222	9,200	9,294	88	66	161
Primary care management	11,169	11,337	11,281	11,470	167	112	300
Ongoing diabetes management (excluding above)	6,833	6,865	6,856	6,875	31	23	42
Diagnostic interview	1,271	1,320	5,588	5,840	50	4,317	4,569
Opportunistic screening	107	112	547	574	5	440	467
Depression treatment	3,215	4,666	5,349	7,728	1,451	2,134	4,513
Total cost	41,562	43,165	48,281	51,209	1,603	6,719	9,647

*CP* Current practice, *IHD* Ischaemic heart disease, *MI* Myocardial infarction, *CHF* Congestive heart failure. ^a^Policy 1 = Collaborative care; ^b^Policy 2 = Opportunistic screening; ^c^Policy 3 = both collaborative care and opportunistic screening

### Wider societal benefits

All three policy changes led to a reduction in both the number of informal care days received and the number of days off paid employment due to ill health. Reductions were largest for the combined policy and smallest for the policy of improved opportunistic screening on its own. The estimated QALY loss due to depression was 1,746 for current practice. Each of the policies was associated with a reduction in lost QALYs, with the largest reduction for the combined policy (1,631; 6.6% of the value for current practice).

### Incremental cost effectiveness ratio

Under an incremental analysis, improved opportunistic screening was dominated by collaborative care being both more expensive and less effective. The ICER for collaborative care compared to current practice was estimated at £10,798 per QALY gained, whilst the ICER for the combined policy compared to collaborative care alone was estimated at £68,017 per QALY. Based on these results, the use of collaborative care would be recommended if using the threshold for cost-effectiveness of £13,000 per QALY, which has been estimated to be the current threshold in the NHS [36].

The results of one-way sensitivity analyses indicated that the cost-effectiveness results were most sensitive to the estimated time until relapse, and the hazard ratio for depression affecting diabetes-related complications. Results were robust to the majority of the other parameters varied. Full results of the sensitivity analyses are available in Additional file 1.

The results presented in Table 4 demonstrate that the key drivers for differences in cost between the policies were related to depression diagnosis, screening and treatment. In contrast, differences in QALYs gained were due to improvements in both diabetes and depression outcomes. If only depression outcomes had been considered, then the ICERs compared with usual practice (£17,000, £91,000 and £50,000 for policies 1, 2 and 3 respectively) would have been higher than when considering both diabetes and depression. Hence, if only outcomes relating to depression were considered then the cost-effectiveness of each of the policies would have been under-estimated.

## Discussion

This study examined the potential cost-effectiveness of three policies for individuals with T2DM and depression in England. These policies represented potential service changes: improvements in opportunistic screening for depression; collaborative care; or a combination of the two. Under current practice the modelled cohort experienced 1.5 million diabetes-related complications, with a cost to the healthcare system of £41.6 billion. All three policies reduced both the number of unidentified depressive episodes, and the number of diabetes-related complications. All three policies were associated with an improvement in HRQoL, but also with an increase in health care costs. Assuming a willingness to pay threshold of £13,000 per QALY, only collaborative care was cost-effective.

A strength of this study was that it considered the entire depression pathway of care as experienced by the majority of individuals with T2DM and comorbid depression. This allowed for a comparison of different types of policy change within a single model, and so reduced variation by using a consistent modelling framework for generating cost-effectiveness evidence. The mathematical model was also based on two previously published and validated models, which lends validity to the results presented.

As is normal in healthcare modelling, the mathematical model represents a simplification of reality and the results presented here need to be interpreted in relation to the assumptions used and evidence available. The main limitation with this study was the lack of relevant and robust evidence for many of the parameters. Much of the evidence about the natural history of depression amongst individuals with T2DM came from studies conducted in the Netherlands and the relevance of this evidence to a UK setting is unclear. Evidence for other parameters was obtained from studies of individuals with depression amongst the general population which may not generalize to individuals with depression and T2DM. The results of sensitivity analyses showed that the modelled association between having depression and developing diabetes-related complications was an important determinant of cost-effectiveness. The evidence for this association was limited as it was derived from a single non-randomised study{Lin, 2010 5299 /id}. A further limitation was the lack of probabilistic sensitivity analysis to evaluate the uncertainty in the results. Had such an analysis been undertaken, it would have been possible to perform value of information analyses, to quantify how uncertainty in each of the model inputs contribute to uncertainty in the cost-effectiveness results, and how much it would be worth spending on further research to reduce this uncertainty [37]. However, twenty nine univariate sensitivity analyses were conducted on key model parameters. The results of these sensitivity analyses suggested that the modelled results remained relatively robust to changes in a number of assumptions.

An important aspect of this study was the modelled bi-directional association between having a diabetes-related complication and depression. This association was modelled via hazard ratios. A more realistic representation may have been to explicitly model the causes for the bi-directional association; for example having depression may lead to poorer self-management which may affect risk factors for diabetes-related complications such as control of HbA1c or smoking status. This was not performed for this study due to a lack of robust evidence. The risk of diabetes related complications is affected by long-term changes in the control of HbA1c, and it is uncertain if a short-term benefit due to reducing time with depression will have a substantial effect on this risk of complications. Because of this, an explicit causal model of the bi-directional association may have led to more reliable estimates of the impact of policies aimed at improving depression identification and the treatment of depression. In addition, there remains considerable uncertainty concerning the association between having a diabetes-related complication and developing depression as no studies were identified which quantified the magnitude of this association.

If longitudinal data on the bi-directional association were available, this would help to establish the causal relationships between diabetes-related complications and depression.

Additional research is required to improve the evidence base and hence increase confidence in the results presented. There is a paucity of evidence on the natural history of depression in patients with diabetes and how it is affected by developing a diabetes-related complication. Evidence about the HRQoL of individuals with diabetes and depression is also required, as is stronger evidence about the bi-directional association between having a diabetes-related complication and depression.

To the authors’ knowledge, this is the first economic evaluation of potential changes to the care pathway for individuals with T2DM and depression. The majority of existing models assess the policy implications for single conditions in isolation [14, 16]. An economic evaluation performed to support NICE clinical guidance modelled the cost-effectiveness of collaborative care amongst people with depression and a chronic physical health problem [11]. However, in this analysis collaborative care only had an impact on depression outcomes; the impact on any comorbid diseases was not modelled. The results from the current analyses suggest that the health-economic benefits from any policy change accrue via changes to both conditions (for example improvements in HRQoL due to a reduction in both diabetes-related complications and the number of unidentified episodes of depression). Hence the health-economic benefits suggested by other models may be under-estimated if the impact of multi-morbidity is not taken into account.

## Conclusions

Using the evidence currently available, the results of this research suggest that policies targeted at identifying and treating depression early in patients with diabetes may lead to a reduction in diabetes related complications and depression, which in turn increase life expectancy and improve HRQoL. The policy change of collaborative care was estimated to be cost-effective under the current NHS threshold.

## Additional file

Additional file 1: Section A: Baseline characteristics. Section B: Modelling depression treatment. Section C: Implementing collaborative care for depression treatment. Section D: Sensitivity analyses performed and results. Section E: Further details on the modelling of the bi-directional association between depression and diabetes-related complications. (DOCX 129 kb)

## Abbreviations

HRQoL: Health-related quality of life
ICER: Incremental cost effectiveness ratio
IHD: Ischaemic heart disease
MI: Myocardial infarction
NHS: National healthcare system
NICE: National institute for health and care
QALYs: Quality adjusted life years
T2DM: Type-2 diabetes
UKPDS OMv2: United Kingdom prospective diabetes study outcomes model version 2

## References

[1] Kearns B, Rafia R, Leaviss J, Preston L, Wong R, Brazier J, et al. Whole pathway modelling of interventions for patients with diabetes and depression. Available at: http://www.eepru.org.uk/Mental-Health%282876640%29.htm. Last Accessed 21^st^ Oct 2016.

[2] Garcia MJ, McNamara PM, Gordon T, Kannell WB (1974). Morbidity and mortality in diabetics in the Framingham population: sixteen year follow-up study. Diabetes.

[3] Das-Munshi J, Stewart R, Ismail K, Bebbington PE, Jenkins R, Prince MJ (2007). Diabetes, common mental disorders, and disability: findings from the UK national psychiatric morbidity survey. Psychosom Med.

[4] Naylor C, Parsonage M, McDaid D, Knapp M, Fossey M, Galea A (2012). Long-term conditions and mental health: the cost of co-morbidities. The King’s fund.

[5] Egedeand LE, Simpson K (2003). Epidemiology, treatment and costs of depression in adults with type 2 diabetes. Expert Rev Pharmacoecon Outcomes Res.

[6] Krein SL, Bingham CR, McCarthy JF, Mitchinson A, Payes J, Valenstein M (2006). Diabetes treatment among VA patients with comorbid serious mental illness. Psychiatr Serv.

[7] Mangin D, Heath I, Jamoulle M (2012). Beyond diagnosis: rising to the multimorbidity challenge. BMJ.

[8] Bower P, Macdonald W, Harkness E, Gask L, Kendrick T, Valderas JM (2011). Multimorbidity, service organization and clinical decision making in primary care: a qualitative study. Fam Pract.

[9] Simon GE, Katon WJ, Lin EH, Rutter C, Manning WG, Von KM (2007). Cost-effectiveness of systematic depression treatment among people with diabetes mellitus. Arch Gen Psychiatry.

[10] Richards DA, Hill JJ, Gask L, Lovell K, Chew-Graham C, Bower P (2013). Clinical effectiveness of collaborative care for depression in UK primary care (CADET): cluster randomised controlled trial. BMJ.

[11] National Institute for Health and Care Excellence (2009). Depression with a chronic physical health problem (CG91).

[12] Coventry P, Lovell K, Dickens C, Bower P, Chew-Graham C, McElvenny D (2015). Integrated primary care for patients with mental and physical multimorbidity: cluster randomised controlled trial of collaborative care for patients with depression comorbid with diabetes or cardiovascular disease. BMJ.

[13] Katon WJ, Von KM, Lin EH, Simon G, Ludman E, Russo J (2004). The Pathways Study: a randomized trial of collaborative care in patients with diabetes and depression. Arch Gen Psychiatry.

[14] Hayes AJ, Leal J, Gray AM, Holman RR, Clarke PM (2013). UKPDS outcomes model 2: a new version of a model to simulate lifetime health outcomes of patients with type 2 diabetes mellitus using data from the 30 year united kingdom prospective diabetes study: UKPDS 82. Diabetologia.

[15] Palmer AJ (2013). Computer modeling of diabetes and its complications: a report on the fifth mount hood challenge meeting. Value Health.

[16] Tosh J, Kearns B, Brennan A, Parry G, Ricketts T, Saxon D, et al. Innovation in health economic modelling of service improvements for longer-term depression: demonstration in a local health community. BMC Health Serv Res. 2013;13(1). 10.1186/1472-6963-13-150 PMC3644496 23622353

[17] Health and Social Care Information Centre. National Diabetes Audit. Available at http://content.digital.nhs.uk/nda. Last Accessed 21^st^ Oct 2016.

[18] Ali S, Davies MJ, Taub NA, Stone MA, Khunti K, Ali S (2009). Prevalence of diagnosed depression in south Asian and white European people with type 1 and type 2 diabetes mellitus in a UK secondary care population. Postgrad Med J.

[19] Bot M, Pouwer F, Ormel J, Slaets JP, de Jonge P, Bot M (2010). Predictors of incident major depression in diabetic outpatients with subthreshold depression. Diabet Med.

[20] Nefs G, Pouwer F, Denollet J, Pop V (2012). The course of depressive symptoms in primary care patients with type 2 diabetes: results from the diabetes, depression, type D personality zuidoost-brabant (DiaDDZoB) study. Diabetologia.

[21] Lustman PJ, Clouse RE, Nix BD, Freedland KE, Rubin EH, McGill JB (2006). Sertraline for prevention of depression recurrence in diabetes mellitus: a randomized, double-blind, placebo-controlled trial. Arch Gen Psychiatry.

[22] Bhattarai N, Charlton J, Rudisill C, Gulliford MC (2013). Prevalence of depression and utilization of health care in single and multiple morbidity: a population-based cohort study. Psychol Med.

[23] British Medical Association. Quality and Outcomes Framework for 2012/13: Guidance for PCOs and practices. 1-3-2012. Available at https://www.myhealth.london.nhs.uk/sites/default/files/u1217/gpqofguidance20122013.pdf. Last Accessed 21^st^ Oct 2016.

[24] National Institute for Health and Care Excellence (2009). Depression in adults (update).

[25] Baumeister H, Hutter N, Bengel J. Psychological and pharmacological interventions for depression in patients with diabetes mellitus and depression. Cochrane Database Syst Rev. 2012;(12):January. 10.1002/14651858.CD008381.pub2 PMC11972844 23235661

[26] Lin EH, Rutter CM, Katon W, Heckbert SR, Ciechanowski P, Oliver MM (2010). Depression and advanced complications of diabetes a prospective cohort study. Diabetes Care.

[27] Alva M, Gray A, Mihaylova B, Clarke P (2013). The effect of diabetes complications on health-related quality of life: the importance of longitudinal data to address patient heterogeneity. Health Econ.

[28] Solli O, Stavem K, Kristiansen IS. Health-related quality of life in diabetes: The associations of complications with EQ-5D scores. Health Qual Life Outcomes. 2010;8(1). 10.1186/1477-7525-8-18 PMC2829531 20132542

[29] Klarenbach S, Cameron C, Singh S, Ur E (2011). Cost-effectiveness of second-line antihyperglycemic therapy in patients with type 2 diabetes mellitus inadequately controlled on metformin. Can Med Assoc J.

[30] Marrett E, Radican L, Davies MJ, Zhang Q (2011). Assessment of severity and frequency of self-reported hypoglycemia on quality of life in patients with type 2 diabetes treated with oral antihyperglycemic agents: a survey study. BMC Res Notes.

[31] Kaltenthaler E, Brazier J, De Nigris E, Tumur I, Ferriter M, Beverley C (2006). Computerised cognitive behaviour therapy for depression and anxiety update: a systematic review and economic evaluation. Health Technol Assess.

[32] Clarke P, Gray A, Legood R, Briggs A, Holman R (2003). The impact of diabetes-related complications on healthcare costs: results from the United Kingdom prospective diabetes study (UKPDS study No. 65). Diabet Med.

[33] Huang YF, Wei XM, Wu T, Chen R, Guo AM. Collaborative care for patients with depression and diabetes mellitus: a systematic review and meta-analysis. BMC Psychiatry. 2013;13(1). 10.1186/1471-244X-13-260 PMC3854683 24125027

[34] Policy Research Unit in Economic Evaluation of Health and Care Interventions (EEPRU) (2015). EEPRU - value based pricing.

[35] National Institute for Health and Care Excellence (2013). Guide to the methods of technology appraisal.

[36] Claxton K, Martin S, Soares M, Rice N, Spackman E, Hinde S (2015). Methods for the estimation of the national institute for health and care excellence cost-effectiveness threshold. Health Technol Assess.

[37] Strong M, Oakley JE, Brennan A (2014). Estimating multiparameter partial expected value of perfect information from a probabilistic sensitivity analysis sample a nonparametric regression approach. Med Decis Making.

[38] Spijker J, De Graaf R, Bijl RV, Beekman AT, Ormel J, Nolen WA (2002). Duration of major depressive episodes in the general population: results from the Netherlands mental health survey and incidence study (NEMESIS). Br J Psychiatry.

[39] Health and Social Care Information Centre. Quality and Outcomes Framework. Available at http://content.digital.nhs.uk/qof. Last Accessed 21^st^ Oct 2016.

[40] Curtis L. Unit costs of health and social care 2013. Personal Social Services Research Unit; 2013. Available at http://www.pssru.ac.uk/project-pages/unit-costs/2013/. Last Accessed 21^st^ Oct 2016.

[41] National Audit Office (2012). The management of adult diabetes services in the NHS.

[42] Ghatnekar O, Willis M, Persson U (2002). Cost-effectiveness of treating deep diabetic foot ulcers with promogran in four European countries. J Wound Care.

[43] Bristol Myers-Squibb, AstraZeneca (2013). Dapagliflozin in combination therapy for treating type 2 diabetes: manufacturers submission.

