# Supplementary material for: The cost-effectiveness of changes to the care pathway used to identify depression and provide treatment amongst people with diabetes in England: a model-based economic evaluation
Source: BMC Health Serv Res. 2017 Jan 24;17:78. doi: 10.1186/s12913-017-2003-z (PMC5259945; doi:10.1186/s12913-017-2003-z)
Supplement: Additional file 1: — Section A: Baseline characteristics. Section B: Modelling depression treatment. Section C: Implementing collaborative care for depression treatment. Section D: Sensitivity analyses performed and results. Section E: Further details on the modelling of the bi-directional association between depression and diabetes-related complications. (DOCX 129 kb) [file 12913_2017_2003_MOESM1_ESM.docx]

**Additional file 1**

Table of Contents

[A. Baseline characteristics. 2](#_Toc465417273)

[B. Modelling depression treatment. 5](#_Toc465417274)

[C. Implementing collaborative care for depression treatment 11](#_Toc465417275)

[D. Sensitivity analyses performed and results. 13](#_Toc465417276)

[E. Further details on the modelling of the bi-directional association between depression and diabetes-related complications 18](#_Toc465417277)

# Baseline characteristics.

Baseline characteristics are reported in Table SM1. Where possible evidence was taken from the National Diabetes Audit (NDA)[1]. UKPDS baseline characteristics were used when evidence was not available from the NDA. In addition to these baseline characteristics, the following prevalence rates were required for the economic model at baseline:

- Prevalence of individuals with no history of depression (and no current depression)
- Prevalence of individuals with a history of depression but no current depression
- Prevalence of individuals with “current” minor depression on treatment
- Prevalence of individuals with “current” major depression untreated.

Neither the NDA nor the UKPDS reported the prevalence of individuals with T2DM who have depression in England. Instead, data were used from a study carried out in England. Based on an electronic hospital database, out of 4,781 patients with T2DM, 435 (9.3%) had depression. Depression was based on either case documentation or use of antidepressant medication at the therapeutic dose, so is likely to relate to major depression. No English data were available on the prevalence of minor depression (either amongst individuals with T2DM or amongst the general population), so it was assumed that this was the same as the prevalence of major depression. Hence for the economic model, 9.3% of individuals had major depression at baseline, and 9.3% had minor depression at baseline. This value was varied in sensitivity analyses by doubling and halving it.

It was further assumed that amongst individuals without existing depression (at baseline), 9.3% would have a history of depression. This 9.3% was split equally between minor and major depression, giving a baseline history of 4.65% for both. [2]

Amongst individuals with depression at baseline a proportion will be identified (and hence treated). As no published evidence was available for these proportions, they were assumed to increase with severity of depression. The following values for identified (treated) depression were used: 60% for minor, 80% for major. Individuals with current depression (minor or major) were assigned to treatment by assuming that they were at Step ‘1’ of treatment (see Section 5.11.2) for more details.

**Table SM1: Parameters used for the baseline characteristics**

| **Variable** | **Value** | **Source** |
| --- | --- | --- |
| Mean age | 66.48±12.96 | Derived from NDA [1] |
| Mean time with diabetes | 6.37 | Derived from NDA [1] |
| Probability Female | 0.444 | NDA [1] |
| Probability Afro-Caribbean | 0.044 | NDA [1] |
| Probability Indian | 0.130 | NDA [1] |
| Probability Smoke | 0.155 | NDA [1] |
| Mean body mass index | 30.34±7.09 | NDA [1] |
| Mean HbA1c | 8.2±1.5 | UKPDS [3] |
| Mean systolic blood pressure | 143±20 | UKPDS [3] |
| Mean high-density lipoprotein | 1.19±0.3 | UKPDS [3] |
| Mean low-density lipoprotein | 3±0.6 | UKPDS [3] |
| Mean Heart Rate | 72±12 | UKPDS [3] |
| Mean eGFR | 77.5±15 | UKPDS [3] |
| Probability Albuminuria | 0.17 | UKPDS [3] |
| Probability Atrial Fibrillation | 0.005 | UKPDS [3] |
| Probability Peripheral Vascular Disease | 0.14 | UKPDS [3] |
| Mean white blood cell count | 6.8±1.8 | UKPDS [3] |
| Mean haemoglobin | 145±13 | UKPDS [3] |
| Congestive heart failure history | Logistic regression model; see reference | NDA [1] |
| Ischaemic heart disease history | Logistic regression model; see reference | NDA [1] |
| Myocardial infarction history | Logistic regression model; see reference | NDA [1] |
| Stroke history | Logistic regression model; see reference | NDA [1] |
| Blind history | Logistic regression model; see reference | NDA [1] |
| Ulcer history | 0.002 | UKPDS [3] |
| Amputation history | Logistic regression model; see reference | NDA [1] |
| Renal failure history | Logistic regression model; see reference | NDA [1] |
| Probability Minor depression | 9.3% | Ali et al 2009[2] |
| Probability Major depression | 9.3% | Ali et al 2009[2] |
| Probability depression is treated  Minor  Major | 60%  80% | Assumptions |
| Probability history of depression:  Minor  Major | 4.65%  4.65% | Assumptions |

# Modelling depression treatment.

Individuals with T2DM and depression in the economic model could receive any of the following treatments for depression:

- Watchful waiting (or active monitoring): this is based on NICE guidance and constitutes a discussion between the patient and GP about the problem, with information about depression. A follow-up assessment at two weeks is arranged. If the patient has not improved at this follow-up assessment, then patients receive an active treatment.
- Pharmacotherapy: this includes any type of antidepressant medication.
- Low-intensity psychological interventions (psychotherapy): these can take a variety of forms, but can generally be categorised into four groups: structured physical activity, guided self-help based on CBT, peer (self-help) support groups, and computerised CBT.
- High-intensity psychological interventions (psychotherapy): these include: CBT, interpersonal psychotherapy, couple therapy, and counselling.

A schematic of how stepped care treatment for depression is implemented within the economic model is presented in Figure SM1.

**Figure SM1: Schematic of the depression treatment sub-model**


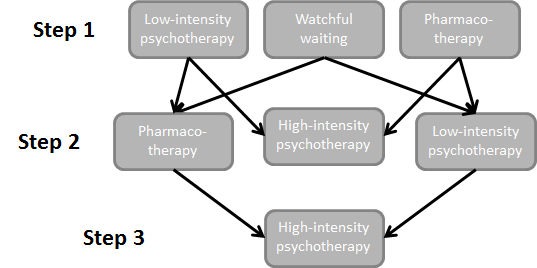


The following duration of treatment were assumed (Table SM2) if treatment was completed

- 2 weeks for watchful-waiting
- 8 weeks for antidepressant therapy based on Tosh et al (2013)[4]
- 22 weeks for low intensity psychotherapy based on Tosh et al (2013)[4]
- 37 weeks for high intensity psychotherapy based on Tosh et al (2013)[4]

The average waiting time for low and high intensity psychotherapy was taken from Richards *et al* (2010).[5] The authors examined the design and implementation of a stepped care model of treatment for common mental health problems across four NHS primary care sites from September 2006 until April 2008. They reported the average waiting times from referral to assessment across all steps and all sites to be 21.8 days (range: 0-224 days).

The waiting time for antidepressant was assumed to be a week. Furthermore, it was assumed that patients who did not respond to antidepressant remain on antidepressants when they step up to low or high intensity psychotherapy. Patients at step 3, who do not response to treatment, are assumed to remain on treatment at step 3 for a maximum duration of 3 years.

**Table SM2: Duration of treatment and waiting time to start treatment assumed in the economic model**

| **Duration of treatment (completers)**   - Watchful-waiting - Antidepressant - Low intensity psychotherapy - High intensity psychotherapy | 2 weeks  8 weeks  22 weeks  37 weeks | Assumption  Tosh et al (2013) [4] |
| --- | --- | --- |
| **Waiting list**   - Watchful-waiting - Antidepressant - Low intensity psychotherapy - High intensity psychotherapy | 0  7 days  21.8 days  21.8 days | Richards et al (2010) [5]  Assumption |

Due to a lack of evidence on the distribution of treatment received, at step 1 in England it was assumed that for minor depression 90% would receive watchful waiting, with the remaining 10% split equally between pharmacotherapy and low-intensity psychotherapy. For major depression it was assumed that 70% would receive pharmacotherapy, 20% would receive low-intensity psychotherapy and 10% would receive watchful waiting. At step 2 the following assumptions were made:

- Individuals receiving watchful-waiting, moved to low-intensity psychotherapy (30%) or pharmacotherapy (70%) irrespective of severity of depression, if they did not spontaneously recover.
- 100% of patients with minor depression who did not respond to pharmacotherapy would be stepped up to low-intensity psychotherapy
- 68% and 32% of patients with major depression who did not respond to pharmacotherapy would move to low intensity and high intensity psychotherapy respectively
- 100% of patients who did not respond to low-intensity psychotherapy would be stepped-up to high-intensity psychotherapy.

***Evidence on response to treatment***

The scoping search of reviews identified two systematic reviews that reported synthesised treatment effects for both pharmacological and psychological treatments for major depression amongst people with diabetes. One review was by Van Der Feltz-Cornelius *et al* (2010) [6], the other was a Cochrane review (Baumeister et al., 2012[7]). Evidence from the Baumeister review [7] was preferred as this included more recent studies; outcome measures on depression remission, and could be used directly in the economic model. Further details on this study are available in section 2.2. Using meta-analysis, the study reported an OR for remission of 2.50 for pharmacological treatment compared with no treatment and 2.88 for psychological treatment compared with no treatment.

Response rates for each treatment were calculated by applying published effect sizes to the probability of spontaneously responding (recovering) in the absence of treatment. NICE clinical guidance on depression with a chronic physical health problem [8] uses a spontaneous recovery rate of 20% for individuals with major depression. This value was based on the expert opinion on the guideline development group.

None of these reviews reported the effectiveness for high-intensity psychotherapy for major depression amongst people with diabetes. The effect size for low-intensity psychotherapy was adjusted using the relative effectiveness between high and low-intensity psychotherapy (RR = 2.25) reported by Tosh et al (2013) [4]. The treatment effectiveness data used in this study was based on local IAPT data.

No study was identified which provided evidence on the effectiveness of treatments for individuals with minor depression and diabetes. Instead, due to the lack of identified evidence, evidence from the general population reported in the NICE CG90 [9] for pharmacological and psychological intervention was used. The study reported a RR of 1.01 for pharmacological treatment compared with no treatment and a RR of 1.16 for psychological treatment compared with no treatment.

As for major depression, the effect size for low-intensity psychotherapy for minor depression was adjusted using the relative effectiveness between high and low-intensity psychotherapy (RR=2.25) reported by Tosh et al (2013) [4].

***Evidence on drop out***

No robust evidence was identified on the probability of dropping out from treatment for individuals with major depression and T2DM. In addition, No study was identified which provided evidence on the drop-out rates for individuals with minor depression and diabetes. Instead, due to the lack of identified evidence, evidence from the general population reported in the NICE clinical guidance 90 [12] for pharmacological and psychological intervention was used. For pharmacological interventions, 55 individuals out of 187 dropped-out, giving a drop-out rate of 31.55%. Hence the drop-out rate for minor depression for pharmacotherapy (31.55%) was used for both minor and major depression in the model. It was further assumed that the drop-out rate for low-intensity psychotherapy would be 30% for both minor and major depression.

No studies reported drop-out rates from high-intensity psychotherapy for either major or minor depression amongst people with diabetes. Hence it was assumed that this drop-out rate would be the same as from low-intensity psychotherapy (30%).

For watchful-waiting, patients were able to drop-out if they did not return for assessment at two weeks. No evidence was identified during our rapid searches. A 10% and 35% drop out rate for patients with minor and major depression respectively were assumed.

The probability of dropping-out was varied in sensitivity analyses; for one the probability was set to 15% for all treatments and for the other it was set to 45%.

The treatment effectiveness data used within the economic model are summarised in Table SM3.

**Table SM3: Treatment effectiveness data used within the economic model**

|  | Minor depression | Major depression | Source |
| --- | --- | --- | --- |
| Probability of remission under no treatment | 20% | | NICE CG91 [8] |
| Effect size for response compared to no treatment:  Pharmacotherapy  Low-intensity psychotherapy | RR= 1.01  RR= 1.16 | OR= 2.50  OR= 2.88 | NICE CG90 [8] for minor  Baumeister (2012) [7] for major |
| Probability of responding to pharmacotherapy | 20.18% | 38.46% | Derived from above |
| Probability of responding to low-intensity psychotherapy | 22.81% | 41.86% | Derived from above |
| Effect size for response for high-intensity compared to low-intensity psychotherapy | RR= 2.25 | | Tosh *et al* (2013) [4] |
| Probability of responding to high-intensity psychotherapy | 44.14% | 61.83% | Derived from above |
| Probability of dropping-out  Pharmacotherapy  Low-intensity psychotherapy  High- intensity psychotherapy  Watchful waiting | 31.55%  30.00%  30.00%  10% | 31.55%  30.00%  30.00%  35% | NICE CG90 [8]  Baumeister (2012) [7]  Assumption  Assumption |

RR: Relative Risk; OR: Odds Ratio

***Evidence on resource use and costs***

Resource use relating to depression treatment are provided in Table SM4. Data on the number of IAPT appointments was taken from the previous economic evaluation of Tosh et al[4], which used observational data. Data on the number of primary care appointments required was based on assumptions. In addition, it was assumed that psychotherapy is given through IAPT services. This is a simplification as in reality IAPT may not be available to everyone in England.

**Table SM4: Summary of resource used in the economic model**

| **Resource use** | **Basecase** | **References** |
| --- | --- | --- |
| Structured interview | 1 primary care appointment | Assumption |
| Number of primary care appointment for patients on antidepressant (initial treatment) | 3 if complete (responder/non-responder)  1 if drop out | Tosh et al (2013) [4] |
| Number of primary care appointment for patients receiving watchful-waiting (completers) | 2 if complete (responder/non-responder)  1 if drop-out | Assumption |
| Number of low intensity psychotherapy session for completers responder/non-responder) | 3 IAPT +1 assessment  2 primary care appointments | Tosh et al (2013) [4] and assumption |
| Number of low intensity psychotherapy session if drop out | 1 IAPT +1 assessment  1 primary care appointment | Tosh et al (2013) [4] and assumption |
| Number of high intensity psychotherapy session for completers responder/non-responder) | 7 IAPT +1 assessment  2 primary care appointments | Tosh et al (2013) [4] and assumption |
| Number of high intensity psychotherapy session if drop out | 3 IAPT +1 assessment  1 primary care appointment | Tosh et al (2013) [4] and assumption |

# Implementing collaborative care for depression treatment

Although collaborative care is recommended by NICE clinical guidelines for individuals with depression and a long-term chronic physical health problem [8], it is not yet routinely implemented within the United Kingdom. Because of this, the additional resource use associated with collaborative care were based on resource use patterns described in NICE CG91 [8]. The effectiveness of collaborative care was taken from a recently published meta-analysis by Huang et al (2013) [10]. The analysis included eight studies containing 2,238 patients. The authors reported that collaborative care was associated with a significant improvement in depression treatment response (relative risk [RR] =1.33, 95% CI =1.05-1.68), depression remission (adjusted RR =1.53, 95% CI =1.11-2.12) and higher rates of adherence to antidepressant medication (RR = 1.79, 95% CI =1.19-2.69). In the economic model, it was assumed that the RR for response and non-drop-out rate for patients with collaborative care was 1.33 and 1.79 respectively compared with usual care.

In NICE CG91 it was assumed that the case manager in the collaborative approach co-ordinates care and is in face to face contact or telephone contact with the service use 10 times over the 6 month period of treatment and has three contacts over the 6 months maintenance period. It was also assumed that the case manager liaises with the GP and that the case manager will undergo supervision by a senior mental health professional. An average of 8 minutes for contact over 3 months for liaison with the GP at a cost of £0.47 per minute was assumed. Furthermore, 2 minutes supervision time per patient was assumed for a 30 to 35 patient caseload at a cost of £0.47 per minute.

Based on these estimates, the number of telephone contacts, face to face contacts, liaison time with GP and supervision time and costs assumed in the economic model are summarised in Table SM5, for patients on antidepressants, low psychotherapy and high psychotherapy, for completers (responders/non-responders). Individuals dropping out of treatment were assumed to incur half the cost. Unit costs were taken from the NICE CG91[8]. A cost of £33 per hour of client contact was assumed for face to face contact, £28 for telephonic contact. The cost associated with liaison with GP and supervision by a psychiatrist was assumed to be £0.47 and £0.47 respectively. The role of case manager does not currently exist within England, so the costing used in the NICE CG91 economic model [8] was used.

**Table SM5: Estimated resource use and costs associated with the addition of a case manager**

|  | Telephone  contact | Face to face  contact  (1 hour) | Face to face  contact  (30 minutes) | Liaison with  GP  (minutes) | Supervision  by a psychiatrist  (minutes) | Total cost |
| --- | --- | --- | --- | --- | --- | --- |
| Antidepressant | | | | | | |
| Completers | 3 | 1 | 1 | 8 | 8 | £85 |
| Drop-out |  |  |  |  |  | £43 |
| Low psychotherapy | | | | | | |
| Completers | 8 | 1 | 1 | 16 | 22 | £142 |
| Drop-out |  |  |  |  |  | £71 |
| High psychotherapy | | | | | | |
| Completers | 12 | 2 | 2 | 24 | 37 | £240 |
| Drop-out |  |  |  |  |  | £120 |

# Sensitivity analyses performed and results.

A number of univariate sensitivity analyses were conducted on key model parameters to determine if the model results were robust to variations in the values used. The following parameters were varied.

In the base-case, it was assumed that 5% (15%) of GP appointments included a screen for patients with no history of depression (patients with a history of depression). These values were varied in sensitivity analysis assuming that the rates were doubled and halved.

Due to the lack of published evidence, the incidence of depression was assumed to be the same for minor and major depression in the base-case. In reality, it is likely that the incidence for minor depression is higher than the incidence for major depression. A sensitivity analysis was conducted assuming the incidence of minor depression to be double that for major depression. Two further sensitivity analyses were conducted; one which halved the incidence of major depression and one which halved the incidence of both types of depression.

It was believed that individuals with diabetes and depression are less likely to attend primary care appointments than individuals with diabetes alone (excluding appointments associated with their depression treatment). Consequently, in the base-case, it was assumed that individuals with minor and major depression had 8 primary care appointments a year (other than those associated with their management of depression). I.e. a reduction of around 35% of attending primary care appointments compared with non-depressed individuals. A sensitivity analysis was conducted assuming the same number of primary care appointments as for non-depressed individuals. A further sensitivity analysis assumed that individuals with depression had 4 primary care appointments a year.

There were also uncertainty on the link between diabetes related complications and depression, and the link between depression and diabetes related complications. In the base-case, a 50% increase (RR: 1.5) in the risk of developing depression was assumed if an individual experienced a diabetes related complication. This value was varied in sensitivity analysis assuming the risk of developing depression was tripled (RR: 3), was not present (RR: 1), was only present for microvascular complications and was only present for macrovascular complications. The link in the other direction (depression increasing the probability of a diabetes complication) was also varied; one sensitivity analysis assumed no link (RR = 1), another assumed a RR of 1.5 for major and minor, and another assumed a RR of 2 for major and minor.

In the base-case it was assumed that opportunistic screening occurred at an additional cost of £2. Two sensitivity analyses were conducted, one assuming a cost of £4 for screening the other no cost.

There was also uncertainty on the impact of depression on quality of life. In the base-case the absolute decrements in utility used was -0.3 for major depression. Two sensitivity analyses were conducted, one using an absolute decrement of -0.1 for major depression, the other using a decrement of -0.5 for major depression.

The baseline prevalence of depression and the time to relapse were also varied in sensitivity analyses by doubling and halving their base-case values.

Four sensitivity analyses were conducted regarding depression treatment. In one sensitivity analysis, all individuals with major depression received antidepressant medication as first-line treatment. In another analysis, all individuals received antidepressant medication after stepping-up from watchful waiting. Two further sensitivity analyses were performed, one set all drop-out rates from depression treatment to 15%, the other set them to 45%.

Two sensitivity analyses were performed that examined the annual diabetes review. In one analysis it was assumed that everyone attended the annual review and that this review always included a depression screen for individuals who were not currently receiving depression treatment. In a separate sensitivity analysis the probability that the annual review includes a depression screen was set to 60%.

Another sensitivity analysis used values of 98% and 86% for the sensitivity and specificity of the opportunistic screen (respectively).

A final sensitivity analysis explored the impact of using a shorter (ten-year) time horizon on the cost-effectiveness results.

Results of the 30 univariate (one-way) sensitivity analyses conducted are displayed in Table SM6. Calculated ICERs are all relative to current practice.

**Table SM6. Results of sensitivity analyses**

| **Description**  **(Costs 2013 UK per £1,000,000. QALYs per 1,000)** | | **Current practice** | **Policy 1** | **Policy 2** | **Policy 3** |
| --- | --- | --- | --- | --- | --- |
| Base-case results | Costs | 29,626 | 30,676 | 34,475 | 36,431 |
|  | QALYs | 12,006 | 12,103 | 12,082 | 12,188 |
|  | ICER |  | £10,798 | £63,810 | £37,421 |
| Probability of attending annual review and of receiving a depression screen (if not being treated) = 100% for both. | Costs | 31,041 | 32,223 | 35,750 | 37,558 |
|  | QALYs | 12,013 | 12,116 | 12,099 | 12,192 |
|  | ICER |  | £11,399 | £54,630 | £36,368 |
| Probability that the annual review includes a depression screen = 60% (base-case = 86%) | Costs | 29,393 | 30,470 | 34,495 | 36,389 |
|  | QALYs | 12,009 | 12,098 | 12,089 | 12,188 |
|  | ICER |  | £12,141 | £64,146 | £39,184 |
| Double the time to relapse. | Costs | 29,993 | 31,528 | 33,965 | 35,319 |
|  | QALYs | 11,867 | 11,957 | 12,270 | 12,357 |
|  | ICER |  | £17,155 | £9,875 | £10,872 |
| Halve the time to relapse. | Costs | 29,299 | 29,989 | 35,138 | 37,604 |
|  | QALYs | 12,206 | 12,290 | 11,937 | 12,035 |
|  | ICER |  | £8,233 | -£21,642 | -£48,541 |
| Drop out rates are 15% for all treatments and types of depression (base-case = 30%). | Costs | 30,198 | 31,090 | 35,475 | 37,022 |
|  | QALYs | 12,038 | 12,136 | 12,120 | 12,217 |
|  | ICER |  | £9,085 | £64,101 | £38,039 |
| Drop out rates are 45% for all treatments and types of depression (base-case = 30%). | Costs | 29,072 | 30,412 | 33,717 | 35,978 |
|  | QALYs | 11,973 | 12,073 | 12,046 | 12,147 |
|  | ICER |  | £13,398 | £63,884 | £39,643 |
| Average annual number of GP appointments for people with depression = 12 (base-case = 8). | Costs | 30,773 | 31,880 | 35,360 | 37,177 |
|  | QALYs | 12,029 | 12,125 | 12,090 | 12,189 |
|  | ICER |  | £11,541 | £75,463 | £40,007 |
| Average annual number of GP appointments for people with depression = 4 (base-case = 8). | Costs | 28,245 | 29,208 | 33,378 | 35,398 |
|  | QALYs | 11,994 | 12,069 | 12,081 | 12,190 |
|  | ICER |  | £12,837 | £58,811 | £36,579 |
| Sensitivity of screening test is 98%, specificity is 86% (base-case values = 95% and 66% respectively). | Costs | 29,291 | 30,429 | 32,478 | 34,281 |
|  | QALYs | 12,002 | 12,102 | 12,084 | 12,188 |
|  | ICER |  | £11,339 | £38,808 | £26,807 |
| The incidence of minor depression is doubled. | Costs | 29,780 | 30,900 | 34,775 | 36,949 |
|  | QALYs | 11,935 | 12,035 | 12,019 | 12,134 |
|  | ICER |  | £11,270 | £59,479 | £36,069 |
| The incidence of minor depression is halved. | Costs | 29,449 | 30,476 | 34,460 | 36,126 |
|  | QALYs | 12,097 | 12,194 | 12,170 | 12,257 |
|  | ICER |  | £10,637 | £68,968 | £41,830 |
| The incidence of both minor and major depression is halved. | Costs | 29,389 | 30,226 | 34,190 | 35,683 |
|  | QALYs | 12,174 | 12,254 | 12,236 | 12,321 |
|  | ICER |  | £10,492 | £77,512 | £42,830 |
| First-line treatment is 100% pharmacotherapy. | Costs | 29,533 | 30,695 | 34,468 | 36,445 |
|  | QALYs | 12,017 | 12,118 | 12,101 | 12,211 |
|  | ICER |  | £11,455 | £58,729 | £35,489 |
| Second-line treatment following watchful waiting is 100% pharmacotherapy. | Costs | 29,532 | 30,623 | 34,458 | 36,344 |
|  | QALYs | 12,003 | 12,109 | 12,084 | 12,191 |
|  | ICER |  | £10,344 | £60,577 | £36,173 |
| Screening cost = £0 (base = £2). | Costs | 29,552 | 30,598 | 34,073 | 36,012 |
|  | QALYs | 12,006 | 12,103 | 12,082 | 12,188 |
|  | ICER |  | £10,765 | £59,497 | £35,524 |
| Screening cost = £4 (base = £2). | Costs | 29,701 | 30,754 | 34,877 | 36,850 |
|  | QALYs | 12,006 | 12,103 | 12,082 | 12,188 |
|  | ICER |  | £10,831 | £68,122 | £39,318 |
| Disutility due to major depression = 0.1 (base = 0.3) | Costs | 29,626 | 30,676 | 34,475 | 36,431 |
|  | QALYs | 12,376 | 12,447 | 12,431 | 12,497 |
|  | ICER |  | £14,780 | £87,340 | £56,104 |
| Disutility due to major depression = 0.5 (base = 0.3) | Costs | 29,626 | 30,676 | 34,475 | 36,431 |
|  | QALYs | 11,267 | 11,417 | 11,384 | 11,570 |
|  | ICER |  | £7,017 | £41,466 | £22,461 |
| Hazard ratio for diabetic complications affecting depression = 1 for all. | Costs | 29,448 | 30,587 | 34,507 | 36,370 |
|  | QALYs | 12,017 | 12,111 | 12,096 | 12,194 |
|  | ICER |  | £12,217 | £64,550 | £39,269 |
| Hazard ratio for diabetic complications affecting depression = 3 for all. | Costs | 29,526 | 30,658 | 34,594 | 36,494 |
|  | QALYs | 11,993 | 12,101 | 12,076 | 12,174 |
|  | ICER |  | £10,490 | £60,838 | £38,581 |
| Hazard ratio for depression = 1 for microvascular complications and 1.5 for macrovascular complications. | Costs | 29,624 | 30,700 | 34,503 | 36,393 |
|  | QALYs | 12,006 | 12,115 | 12,092 | 12,191 |
|  | ICER |  | £9,894 | £56,972 | £36,604 |
| Hazard ratio for depression = 1.5 for microvascular complications and 1 for macrovascular complications. | Costs | 29,584 | 30,709 | 34,469 | 36,406 |
|  | QALYs | 12,016 | 12,110 | 12,085 | 12,191 |
|  | ICER |  | £12,014 | £70,500 | £38,976 |
| Hazard ratio for depression affecting diabetic complications = 1 for all. | Costs | 29,412 | 30,524 | 34,479 | 36,403 |
|  | QALYs | 12,101 | 12,159 | 12,145 | 12,229 |
|  | ICER |  | £19,223 | £115,115 | £54,668 |
| Hazard ratio for depression affecting diabetic complications = 1.5 for all. | Costs | 29,312 | 30,519 | 34,205 | 36,152 |
|  | QALYs | 11,787 | 11,935 | 11,907 | 12,050 |
|  | ICER |  | £8,160 | £40,773 | £26,062 |
| Hazard ratio for depression affecting diabetic complications = 2 for all. | Costs | 29,230 | 30,339 | 34,056 | 35,935 |
|  | QALYs | 11,502 | 11,723 | 11,682 | 11,874 |
|  | ICER |  | £5,012 | £26,719 | £17,990 |
| Prevalence of depression (minor and major) doubled. | Costs | 29,856 | 31,020 | 34,693 | 36,869 |
|  | QALYs | 11,901 | 12,000 | 11,987 | 12,091 |
|  | ICER |  | £11,775 | £56,141 | £36,900 |
| Prevalence of depression (minor and major) halved. | Costs | 29,449 | 30,462 | 34,389 | 36,196 |
|  | QALYs | 12,071 | 12,154 | 12,150 | 12,237 |
|  | ICER |  | £12,304 | £63,177 | £40,702 |
| Halve cost of collaborative care | Costs | 29,626 | 30,241 | 34,475 | 35,710 |
|  | QALYs | 12,006 | 12,103 | 12,082 | 12,188 |
|  | ICER |  | £6,325 | £63,810 | £33,457 |
| Double cost of collaborative care | Costs | 29,626 | 31,546 | 34,475 | 37,873 |
|  | QALYs | 12,006 | 12,103 | 12,082 | 12,188 |
|  | ICER |  | £19,744 | £63,810 | £45,349 |
| Prevalence of depression (minor and major) halved. | Costs | 29,626 | 30,676 | 34,475 | 36,431 |
|  | QALYs | 12,006 | 12,103 | 12,082 | 12,188 |
|  | ICER |  | £10,798 | £63,810 | £37,421 |
| Ten-year time horizon | Costs | 19,583 | 20,143 | 22,989 | 24,099 |
|  | QALYs | 8,286 | 8,327 | 8,315 | 8,375 |
|  | ICER |  | £13,583 | £116,137 | £50,566 |

Policy 1 = Collaborative care; Policy 2 = Opportunistic screening; Policy 3 = both collaborative care and opportunistic screening. ICER = incremental cost-effectiveness ratio. QALYs = Quality adjusted life-years.

# Further details on the modelling of the bi-directional association between depression and diabetes-related complications

***Example of how successful depression treatment may shorten the length of a depressive episode***

Successful treatment may not result in a shorter time to recovery. If an individual was due to spontaneously recover from their depression during their treatment, then successful treatment would not have affected the amount of time spent with depression. This is depicted in Figure SM2, which shows the two possible outcomes for people who respond to treatment. In the top example, the treatment brings-forwards the time to recovery from time ‘b’ to time ‘a’. In the bottom example treatment does not alter the time to recovery. Treatment costs are the same in both examples.

**Figure SM2: Relationship between time spent receiving depression treatment and time spent with depression**


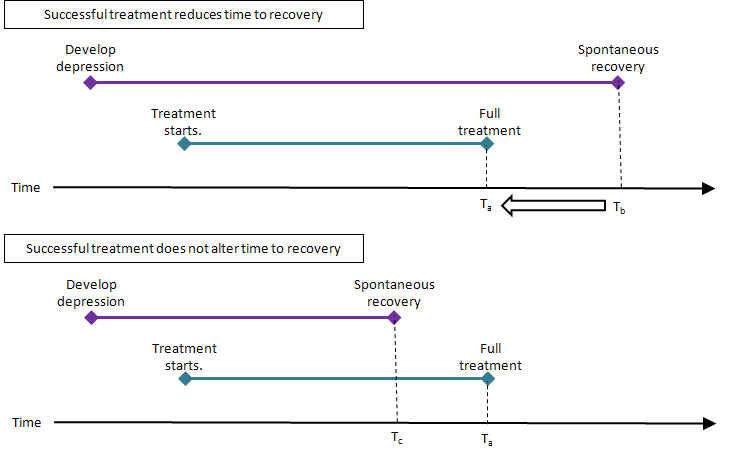


***Example of how developing a diabetes-related complication will decrease the time to developing depression.***

A schematic of the relationship between developing a diabetic complication and time to depression is provided in Figure SM3. In this figure, a diabetes-related complication occurs (at time T=a). ‘E’ is the effect size of developing depression due to developing a complication. For example, if E=2, the remaining time to developing depression is halved. It should be noted that having a complication does not impact on length of depression or time to progression (due to a lack of data).

**Figure SM3: Relationship between developing a diabetes-related complication and developing depression**


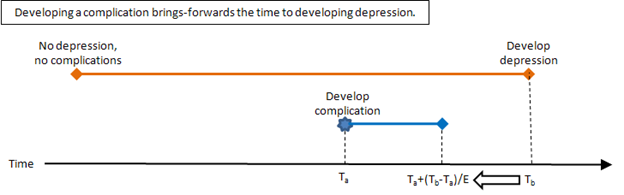


Reference List

1. Health and Social Care Information Centre. National Diabetes Audit. Available at <http://www.hscic.gov.uk/nda> . 2013. Last accessed 21^st^ October 2016.

2. Ali S, Davies MJ, Taub NA, Stone MA, Khunti K, Ali S *et al*.: **Prevalence of diagnosed depression in South Asian and white European people with type 1 and type 2 diabetes mellitus in a UK secondary care population.** *Postgraduate Medical Journal* 2009, **85:** 238-243.

3. Hayes AJ, Leal J, Gray AM, Holman RR, Clarke PM: **UKPDS Outcomes Model 2: a new version of a model to simulate lifetime health outcomes of patients with type 2 diabetes mellitus using data from the 30 year United Kingdom Prospective Diabetes Study: UKPDS 82.** *Diabetologia* 2013 **56**(9), 1925-1933.

4. Tosh J, Kearns B, Brennan A, Parry G, Ricketts T, Saxon D *et al*.: **Innovation in health economic modelling of service improvements for longer-term depression: demonstration in a local health community.** *BMC Health Services Research* 2013, **13**(1)

5. Richards DA, Weaver A, Utley M, Bower P, Cape J, Gallivan S *et al*.: **Developing evidence based and acceptable stepped care systems in mental health care: an operational research project.** *Final Report, NIHR Service Delivery and Organisation Programme* 2010.

6. van der Feltz-Cornelis CMN: **Effect of interventions for major depressive disorder and significant depressive symptoms in patients with diabetes mellitus: A systematic review and meta-analysis.** *General Hospital Psychiatry* 2010, **32:** 380-395.

7. Baumeister H, Hutter N, Bengel J: **Psychological and pharmacological interventions for depression in patients with diabetes mellitus and depression.** *Cochrane Database of Systematic Reviews* 2012, **12:** January.

8. National Institute for Health and Care Excellence. Depression with a chronic physical health problem (CG91). October 2009. Available at https://www.nice.org.uk/guidance/cg91. Last accessed 21^st^ October 2016.

9. National Institute for Health and Care Excellence. Depression in adults (update). October 2009. Available at https://www.nice.org.uk/guidance/cg90 Last accessed 21^st^ October 2016.

10. Huang YF, Wei XM, Wu T, Chen R, Guo AM: **Collaborative care for patients with depression and diabetes mellitus: a systematic review and meta-analysis.** *BMC Psychiatry* 2013, **13**.
